# Dysregulation of CXCL9 and reduced tumor growth in Egr-1 deficient mice

**DOI:** 10.1186/1756-8722-2-7

**Published:** 2009-02-07

**Authors:** Giuseppe Caso, Catherine Barry, Gerald Patejunas

**Affiliations:** 1 Department of Surgery, Stony Brook University, Stony Brook, NY, USA; 2 Abbott Laboratories, Des Plaines, IL, USA

## Abstract

**Background:**

Early growth response-1 (Egr-1) is an immediate-early transcription factor inducible in the vasculature in response to injury, shear stress, and other stimuli. Mice lacking Egr-1 have a profound deficit in the ability to recover from femoral artery ligation, suggesting a role in neovascularization. Previous studies have shown that manipulating Egr-1 expression can have either positive or negative effects on tumor growth. We hypothesized that Egr-1 knockout mice might exhibit reduced tumor growth, possibly due to a reduced capacity to respond to angiogenic signals from a growing tumor.

**Results:**

We injected 10^6 ^Lewis lung carcinoma (LLC1) cells subcutaneously in the flank of wild type and Egr-1 knockout mice. The average mass of tumors from wild type mice at 12 days after implantation was 413 +/- 128 mg, while those from Egr-1^-/- ^mice was 219 +/- 81 mg (p = 0.001, mean +/- SD). However, sectioning the tumors and staining with anti-CD31 antibodies revealed no difference in the vascularity of the tumors and there was no difference in angiogenic growth factor expression. Expression of the chemokine Mig (CXCL9) was increased 2.8-fold in tumors from knockout mice, but no increase was found in serum levels of Mig. Natural killer cells have a 1.7-fold greater prevalence in the CD45^+ ^cells found in tumors from Egr-1^-/- ^mice compared to those from wild type mice. Immunohistochemical staining suggests that Mig expression in the tumors comes from invading macrophages.

**Conclusion:**

Mice deficient in Egr-1 exhibit reduced growth of LLC1 tumors, and this phenomenon is associated with overexpression of Mig locally within the tumor. There are no obvious differences in tumor vascularity in the knockout mice. Natural killer cells accumulate in the tumors grown in Egr-1^-/- ^mice, providing a potential mechanism for the reduction in growth.

## Background

Growth of a tumor can be significantly influenced by its interactions with the surrounding stromal tissue. Endothelial and immune system cells that invade the tumor affect its rate of proliferation. Chemokines can act to attract cells of the immune system to the site of tumor growth. Monokine induced by interferon-γ (Mig) [1], also known as CXCL9, is a chemokine that attracts T-cells and natural killer (NK) cells [2]. Mig also has angiostatic properties [3]. Overexpression of Mig in tumors can lead to T-cell accumulation, vascular damage, and tumor regression [4,5].

Egr-1 is a zinc-finger transcription factor that is inducible by radiation [6], serum [7], shear stress [8], and other stimuli in a variety of cell types, including tumor cells [9,10]. Previous studies have examined the effects of manipulating Egr-1 in tumors. Overexpression of Egr-1 delivered via adenovirus resulted in reduced tumor growth and diminished expression of angiogenic factors in a mouse model [11]. However, reduction of Egr-1 levels through use of a DNAzyme also resulted in slower tumor growth [12,13]. In some of these studies it was difficult to clearly distinguish the effects of the delivered reagents on tumor versus stromal tissue.

We have previously shown that Egr-1 knockout mice exhibit a defect in arteriogenesis, as illustrated by their greatly reduced capacity to recover hind limb blood flow after femoral artery ligation [14]. We speculated that the absence of Egr-1 in the stromal tissue of mice might have an effect on tumor growth, possibly due to dysregulation of angiogenic signalling. Our present work shows that growth of at least some tumors is slowed in Egr-1 deficient mice, but with no apparent effect on angiogenesis. Instead, Mig accumulates in the tumor, along with NK cells.

## Results

### Lewis lung carcinoma growth is slowed in Egr-1^-/- ^mice

**Figure 1:**
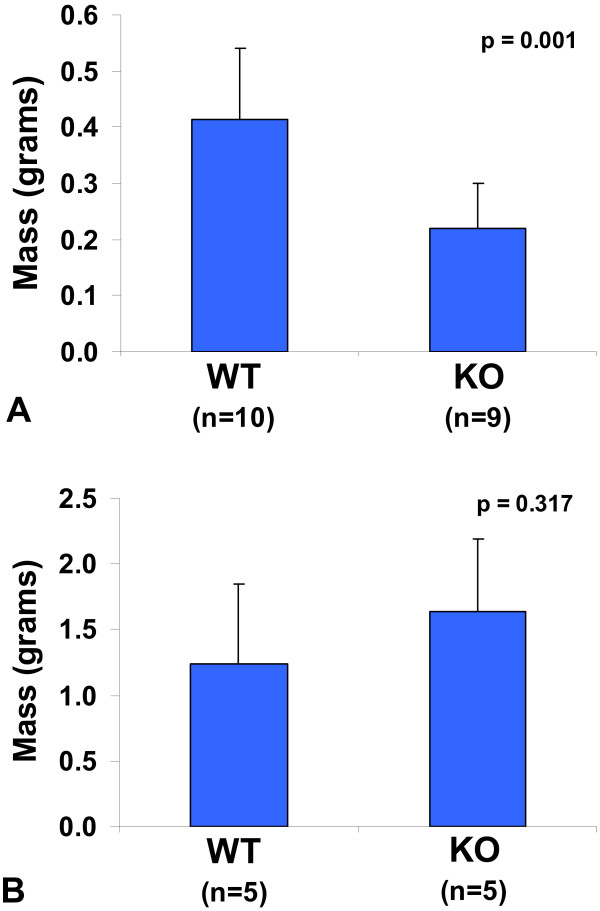
**Weight of tumors grown in wild type and knockout mice**. One million tumor cells were injected subcutaneously in wild type (WT) and Egr-1 knockout (KO) mice. Tumors were excised and weighed after 12 days. Averages and standard deviations are shown, with p values calculated by Student's t-test. (A) Lewis lung carcinoma cells (B) B16F10 melanoma cells.

To assess the rate of tumor growth in Egr-1^-/- ^mice, we introduced 10^6 ^Lewis lung carcinoma cells (LLC1) subcutaneously in the flank of wild type and knockout animals. After 12 days, we excised the tumors and weighed them. Figure 1a shows that tumors from wild type mice are 1.9-fold larger than those from knockout mice (p = 0.001). Repeating this experiment using B16F10 melanoma cells demonstrated no significant difference in the rate of tumor growth between the two types of mice (Figure 1b), as has been previously shown for this cell line [13].

### Mig is overexpressed in LLC1 tumors from Egr-1^-/- ^mice

**Figure 2:**
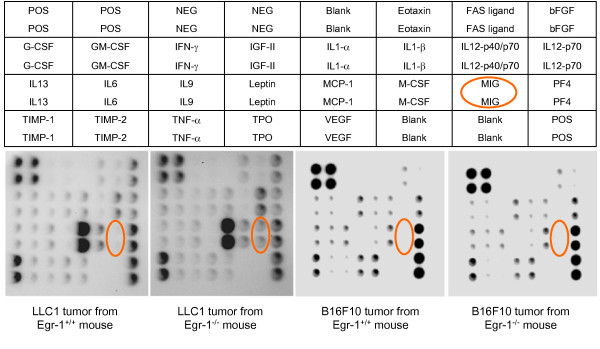
**Antibody array analysis of tumor lysates**. (Top) Schematic of the placement of antibodies on the array. Orange ellipses highlight the position of Mig. POS = positive control, NEG = negative control, bFGF = basic fibroblast growth factor, G-CSF = granulocyte colony stimulating factor (CSF), GM-CSF = granulocyte/macrophage CSF, IGF-II = insulin-like growth factor II, IL = interleukin, MCP-1 = monocyte chemoattractant protein-1, PF4 = platelet factor 4, TIMP = tissue inhibitor of metalloproteinase, TNF = tumor necrosis factor, TPO = thrombopoietin, VEGF = vascular endothelial growth factor. (Bottom, left) arrays treated with LLC1 tumor lysates from wild type and knockout mice. (Bottom, right) arrays treated with B16F10 tumor lysates from wild type and knockout mice.

In an attempt to elucidate molecular differences that might underlie the reduced growth rate in LLC1 tumors, we subjected tumor lysates to an antibody array. The array allows analysis of 24 proteins related to blood vessel growth. We found very little difference in expression patterns between tumors grown in wild type and Egr-1^-/- ^mice, except that Mig was elevated by about 5.8-fold in knockout-derived tumors, and IL-12p40/p70 was elevated about 1.7-fold (Figure 2). Repeating the experiment using lysates from B16F10 tumors failed to show any differences in Mig or IL12p40/p70 expression (Figure 2).

**Figure 3:**
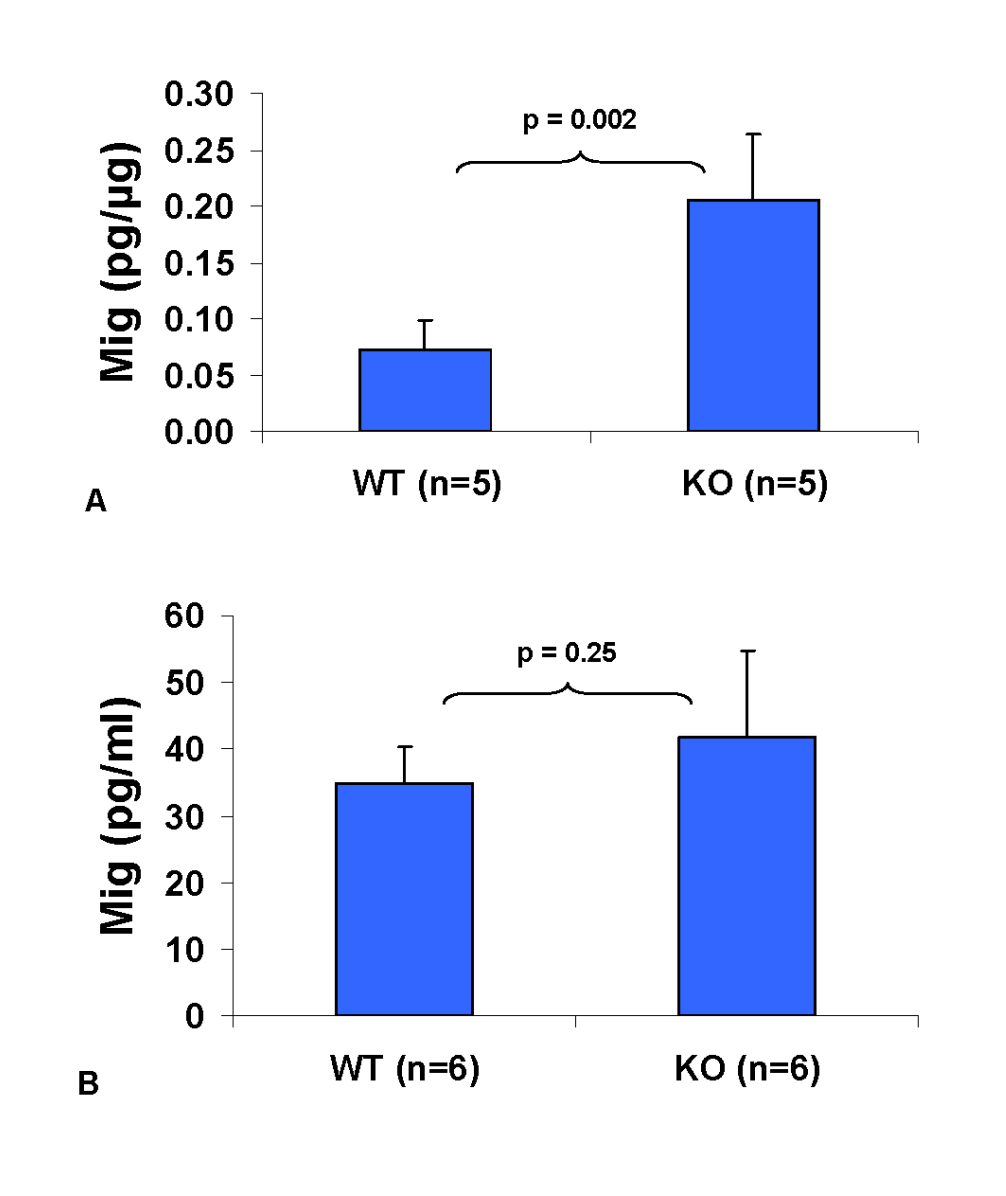
**Confirmation of Mig expression**. Mig was measured using a cytometric bead array. Averages and standard deviations are shown, with p values calculated using Student's t-test. WT = wild type and KO = Egr-1 knockout source animal. (A) Mig in LLC1 tumor lysates, shown as picograms of Mig per microgram of protein. (B) Mig in serum from tumor-bearing mice.

To confirm the expression levels of Mig, we made additional lysates from LLC1 tumors grown for 11–12 days in wild type and Egr-1^-/- ^mice and measured Mig using a BD cytometric bead array. Levels of Mig were 2.8-fold higher in knockout-derived tumors (Figure 3a). To determine whether this disparity represents a systemic difference in Mig expression between the two types of mice, we also measured Mig in serum from the same animals and found no significant difference (Figure 3b). We attempted to measure Mig in the tissue immediately underlying the tumor (peritoneal wall and associated muscle), but the levels were below the threshold of detection of our assay (data not shown).

### Mig is expressed in tumor macrophages in Egr-1^-/- ^mice

**Figure 4:**
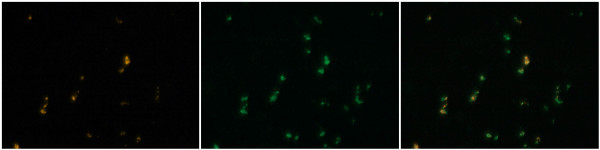
**Colocalization of Mig and CD68 in LLC1 tumor sections**. (Left) Mig staining. (Middle) CD68 staining. (Right) Superposition of the left and middle photos.

Since the tumor Mig does not appear to be derived from serum or surrounding tissue, we hypothesized that it was being made in situ by some type of invading host-derived cell. We sectioned LLC1 tumors after 12 days of growth in knockout mice and performed immunofluorescence staining using antibodies against Mig. We found punctate staining that colocalized with expression of CD68, which is a marker for macrophages (Figure 4).

We attempted to measure Mig in resting monocytes isolated from the spleens of wild type and Egr-1^-/- ^mice using a cytometric bead array, but the levels were below the threshold of detection. Mig is known to be inducible in monocyte/macrophages by interferon-γ (IFN-γ). We cultured splenic monocytes with 100 ng/ml IFN-γ and measured Mig in the supernatant five hours later, but there was no difference in the level of induction between wild type and knockout monocytes (data not shown).

### NK cell invasion of LLC1 tumors in Egr-1^-/- ^mice is greater than in wild type

**Figure 5:**
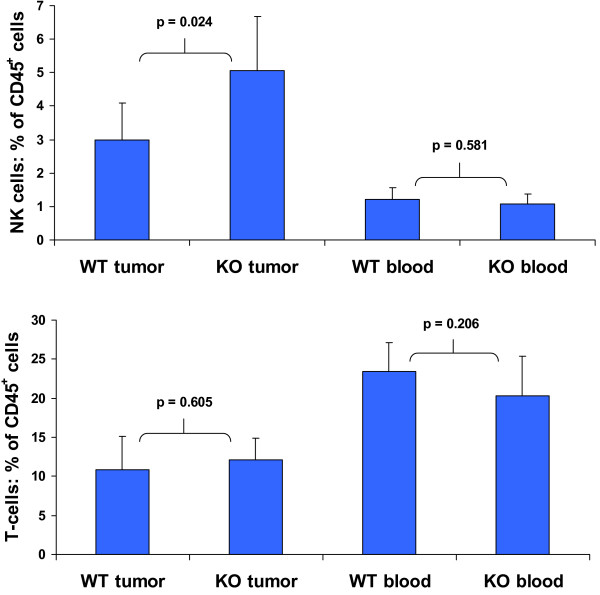
**Prevalence of natural killer (NK) and T-cells**. Cells were labelled and counted by flow cytometry as a percentage of CD45^+ ^cells. Averages and standard deviations are shown, with p values calculated using Student's t-test. WT = wild type and KO = Egr-1 knockout source animal. (Top) NK cells in tumor and whole blood derived from tumor-bearing mice. (Bottom) T-cells in tumor and whole blood derived from tumor-bearing mice.

Mig is known to be chemotactic for T-cells and natural killer (NK) cells [2]. We dissociated LLC1 tumors derived from wild type and Egr-1^-/- ^mice into single cell suspensions and labelled them with fluorescently-tagged antibodies against the T-cell receptor (CD3), leukocyte common antigen (CD45), and NK1.1, a NK cell marker in C57Bl/6 mice. We then counted the number of T-cells and NK cells as a fraction of CD45^+ ^cells in the tumors using flow cytometry. Figure 5 (top panel) shows that there is a significant increase in the percentage of NK cells in tumors derived from knockout mice relative to those from wild type mice. To assess whether the increased numbers of NK cells in the tumors reflects a constitutive property of the knockout mice, we counted cells in whole blood taken from the same animals at the time of tumor harvest. There was no significant difference. We similarly counted T-cells in dissociated tumors and blood and found no significant difference between the wild type and mutant mice (Figure 5, bottom panel). CD11b^+ ^monocyte/macrophages are also similar in number in tumors from the two types of mice (data not shown).

### Capillary growth is normal in LLC1 tumors grown in Egr-1^-/- ^mice

**Figure 6:**
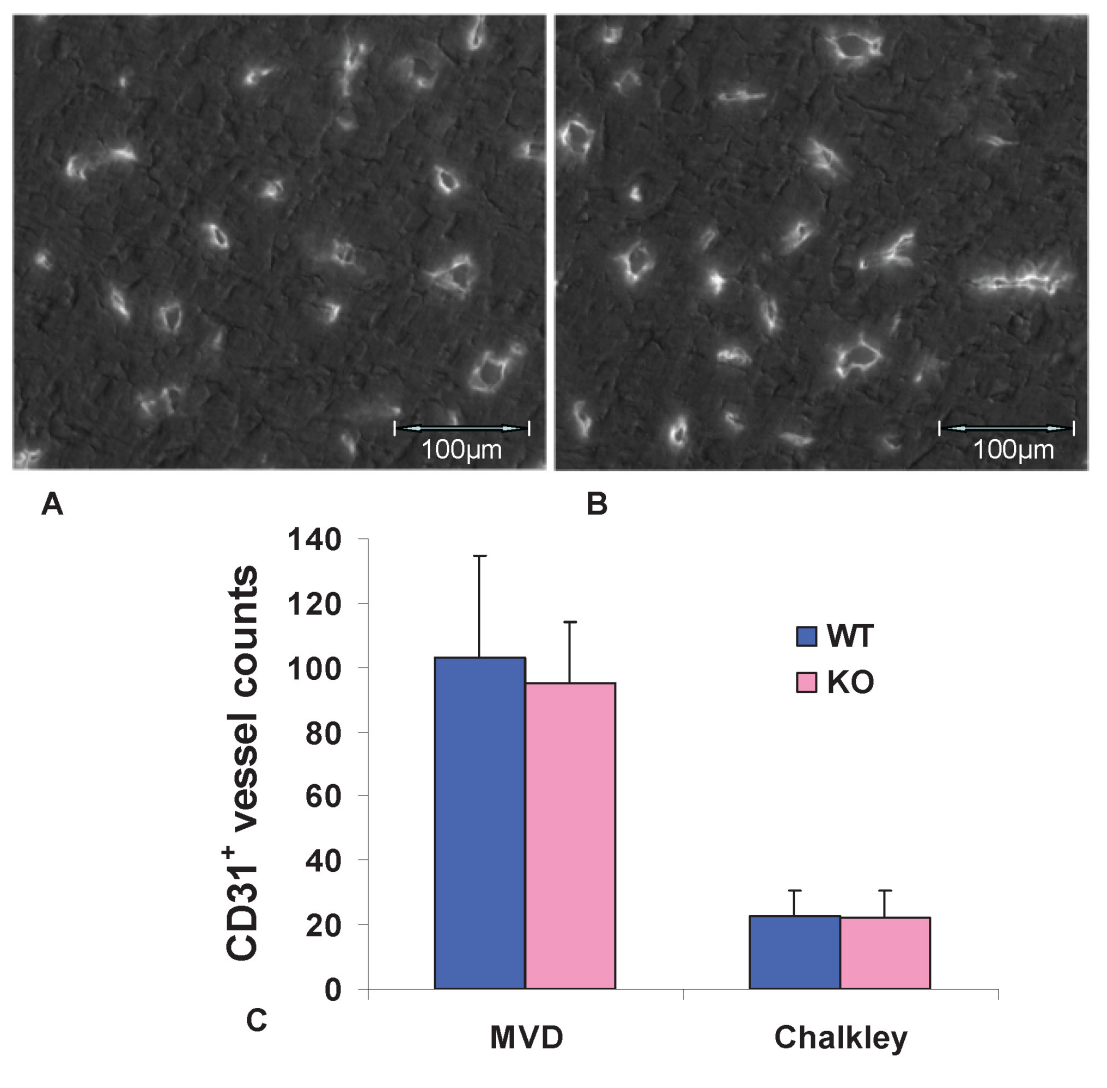
**Vascularity of tumor sections**. LLC1 tumors were sectioned and stained using anti-CD31 antibodies. (A) Section from tumor grown in wild type mouse. (B) Section from tumor grown in Egr-1^-/- ^mouse. (C) Results of blinded counting of sections from three wild type (WT) and Egr-1 knockout (KO) tumors, using either the microvascular density method (MVD), i.e., counting all distinct vessels in a high power field, or the Chalkley method, i.e., placing a gridwork over the photograph and counting those vessels that touch the grid, as described [15]. Values shown are averages and standard deviations.

There is evidence that Mig possesses angiostatic properties [3]. We sectioned LLC1 tumors from wild type and Egr-1^-/- ^mice after 12 days of growth, stained for endothelial cells (Figure 6a, b), and measured vascularity both by the microvascular density method and the Chalkley method [15]. There was no significant difference in the vascularity by either approach (Figure 6c).

## Discussion

Our work demonstrates that the growth of subcutaneous LLC1 tumors in Egr-1^-/- ^mice is impeded. This impediment correlates with overexpression of Mig in the tumor, a phenomenon that is not observed in B16F10 tumors, which do not exhibit slower growth in Egr-1^-/- ^mice. Mig has previously been shown to slow the growth of tumors in various models. In a mouse model of Burkitt's lymphoma, intra-tumoral injection of Mig protein results in partial necrosis of the tumor [5]. Likewise, adenoviral delivery of the Mig gene shrinks non-small cell lung carcinomas [16]. Walser, et al. [4], injected mice with mammary adenocarcinoma cells overexpressing Mig and found that these cells formed smaller tumors than the parental cell line. Our antibody array analysis (figure 2) examined expression of several genes potentially regulated by Egr-1, including bFGF [17], TNF-α [18], IGF-II [19], and M-CSF [20], but there was no significant alteration in expression of these genes between groups.

CXCR3 serves as a receptor for Mig, as well as for related chemokines IP-10 (CXCL10) [21] and I-TAC (CXCL11) [22]. It is expressed on T-cells and NK cells. We were somewhat surprised that there was not a greater degree of T-cell infiltration in the tumors grown in Egr-1^-/- ^mice, but there may have been dysregulation of other chemokines that were not assayed on our antibody array, and these may have influenced the degree of lymphocyte invasion and activation. Also, our analysis looks at one time point, and we cannot exclude the possibility T-cells may be involved at earlier or later time points. While we cannot conclude from our data that NK cells were responsible for the slower tumor growth that we observed, others have implicated NK cells in Mig-mediated tumor inhibition [23] and have shown that NK cells recruited by Mig impair metastasis [4]. Also, Wald, et al. [24] showed that growth of Lewis lung carcinoma tumors is impaired in an NK cell-dependent manner in response to IFN-γ, which stimulates production of Mig.

The connection between the lack of Egr-1 and overexpression of Mig is unclear. We are not aware of any literature suggesting that Egr-1 directly regulates Mig, or whether other Egr family members play a role in its expression. Mig is not produced in the tissue underlying the tumor, nor is it systemically higher in the knockout mice, which suggests that it is being produced in the tumor mass itself. Since the injected tumor cells are identical in the two types of animals, we hypothesized that Mig is produced from a host-derived cell that invades the tumor, and our colocalization experiment with a macrophage marker, CD68, confirms this. Previous studies have shown that monocyte/macrophages develop normally in Egr-1^-/- ^mice [25], and respond to stimulus with lipopolysaccharide similarly to wild type monocytes [26]. We were unable to detect any difference in the expression of Mig in monocytes from knockout mice, but we cannot exclude the possibility that macrophages exposed to the tumor environment may express Mig aberrantly.

We originally hypothesized that there might be a difference in blood vessel growth in the knockout mouse tumors, based on our previous work showing a defect in arteriogenesis in these animals [14]. However, the antibody array revealed no differences in expression of common angiogenic growth factors like VEGF and bFGF. Mig is reported to have an angiostatic effect [3], and disrupted blood vessel growth has been implicated as a factor in the mechanism for Mig-mediated tumor shrinkage in some studies [5,16]. But experiments with breast adenocarcinomas [4] and lung carcinomas [27] have failed to find changes in angiogenesis in Mig-treated tumors. The immunohistochemical staining we employed to measure vascular density did not detect any difference in vascularity, though we cannot rule out subtle effects on vessel growth. Given that Egr-1 can potentially regulate expression of hundreds of genes [28], other factors may have compensated for any angiostatic effects of Mig in our model.

Both over- and under-expression of Egr-1 can impede tumor growth. In a mouse fibrosarcoma model, anti-tumor and anti-angiogenesis effects were observed in response to injection of an adenovirus encoding Egr-1 [11], but the gene was delivered to both tumor and stroma. Other researchers have shown that reducing Egr-1 expression in human breast cancer cells can dampen their growth and invasiveness [12]. Fahmy, et al. [13] used DNAzymes to block expression of murine Egr-1 in nude mice injected with the human breast cancer cell line MCF-7. They found a reduced rate of tumor growth, which they attributed to inhibition of angiogenesis. But since this experiment was performed in athymic nude mice, the role of the immune system is uncertain. Another study examined tumor development in mice genetically predisposed to prostate cancer that had been crossed with Egr-1^-/- ^mice. This study showed a decreased progression of the tumor from carcinoma in situ to invasive carcinoma, though initial growth rate and vascularity were unaffected [29]. Again, both tumor and stroma lacked Egr-1, making it difficult to assess the contribution of these two compartments. Our work stands apart from these previous efforts in that we have looked at the effect of eliminating Egr-1 in the stroma alone using an immunocompetent animal. Doing so has allowed us to uncover a previously undescribed involvement of Egr-1 in Mig regulation and natural killer biology.

A limitation of our study is that our work does not tell us whether Mig is the primary causative agent involved in the reduction of tumor growth seen in the knockout mice. The fact that the B16F10 tumors do not overexpress Mig and also do not exhibit growth inhibition suggests that Mig might be playing a role. The reason for the lack of Mig expression in the B16F10 melanomas is unclear, but we note that the antibody array shows a dramatic difference between the two types of tumors in the expression of monocyte chemoattractant protein-1 (MCP-1). In both wild type and knockout mice, MCP-1 is absent in the melanomas but is so abundant in the LLC1 tumors that it saturates the array. We speculate that the lack of MCP-1 may affect monocyte activity in the B16F10 tumors, and hence Mig expression, but we cannot exclude other potential differences between the two types of tumors.

## Conclusion

We have shown that mice lacking Egr-1 have impaired growth of LLC1 tumors, and that this correlates with increased expression of Mig in the tumor. The Mig appears to come from invading macrophages. Natural killer cells accumulate to a greater extent in the LLC1 tumors of knockout mice compared to those in wild types. There is no obvious difference in vascularity between tumors grown in the two types of mice. Unlike LLC1 cells, B16F10 melanomas exhibit no alteration in Mig or in tumor growth in Egr-1^-/- ^mice, a finding that highlights the importance of the choice of model system when examining tumor/stromal interactions.

## Methods

### Mice and tumor model

Egr-1 knockout mice were obtained from Taconic and maintained on a C57Bl/6 background. Wild type C57Bl/6 mice were used as negative controls. All procedures were approved by the Stony Brook University Institutional Animal Care and Use Committee. Lewis lung carcinoma cells (LLC1) were obtained from ATCC (#CRL-1642) as were B16F10 melanoma cells (#CRL-6475). Both cell lines were maintained in Dulbecco's modified Eagle's medium with 10% fetal bovine serum. One million cells were injected subcutaneously in the flank in a volume of 50 μl of saline. Cells were filtered through a 70 μm filter prior to injection to remove any clumps.

### Flow cytometry

Tumors were excised after 11–12 days of growth, weighed, and digested in 470 units/ml collagenase II and 167 μg/ml hyaluronidase in RPMI medium at 37° for 25 minutes. Single cell suspensions were obtained by trituration and the cells were labelled with antibodies against CD3 (eBioscience, phycoerythrin-labelled), NK1.1 (eBioscience, allophycocyanin-labelled) and CD45 (BioLegend, PerCP-labelled) as described in the text. In some cases, cells were also labelled with anti-CD11b antibodies (BioLegend, Alexa Fluor 488-labelled). After fixation with 10% formalin, cells were analyzed on a FACS Calibur (Becton, Dickinson). Blood cells were similarly measured in whole blood obtained via cardiac puncture from tumor-bearing mice at the time of euthanasia. Blood was cleared of erythrocytes by lysis in ACK lysing buffer (BioWhittaker).

### Expression assays

Lysates were made from powdered frozen tumors and were subjected to analysis on a RayBiotech Mouse Angiogenesis Antibody Array I using the manufacturer's reagents and protocols. Mig levels were measured using a BD Cytometric Bead Array (Becton, Dickinson) on tumor lysates and on serum collected from tumor-bearing mice at the time of euthanasia. Protein in the lysates was measured using the DC protein assay (Bio-Rad).

### Monocyte culture

Mouse spleens were crushed and forced through a 70 μm nylon filter and erythrocytes were lysed with ACK lysing buffer. The resulting cells were labelled with anti-CD11b antibodies (BioLegend, Alexa 488-labelled) and anti-Ly6c antibodies (Southern Biotech, phycoerythrin-labelled). Monocytes were sorted on a FACS Aria (Becton, Dickinson) and cultured in RPMI. IFN-γ was obtained from RayBiotech.

### Immunohistochemistry

Tumors were frozen in optimal cutting temperature (OCT) medium, sectioned, fixed in methacarn, and stained using Alexa 488-labelled anti-CD68 (Serotec) and biotinylated anti-Mig (R&D Systems). The Mig staining was achieved using a tyramide staining kit (Invitrogen). Endothelial cells were stained on frozen sections using biotinylated anti-CD31 (eBioscience) and tyramide staining. Endothelial cell counting was performed by a blinded observer as described [15].

## Competing interests

The authors declare that they have no competing interests.

## Authors' contributions

GC assisted with labeling of cells for flow cytometry and harvesting tumors. CB contributed to the intellectual development of the work and to feasibility studies. GP performed the laboratory and animal work, developed the idea for the project, and wrote the manuscript.
